# Negative linear compressibility in Se at ultra-high pressure above 120 GPa

**DOI:** 10.1107/S2052252522000252

**Published:** 2022-02-01

**Authors:** Shuhua Yuan, Luhong Wang, Sheng-cai Zhu, Fuyang Liu, Dongzhou Zhang, Vitali B. Prakapenka, Sergey Tkachev, Haozhe Liu

**Affiliations:** a Center for High Pressure Science and Technology Advanced Research, Haidian, Beijing 100094, People’s Republic of China; b Center for Interdisciplinary Sciences, Harbin Institute of Technology, Harbin, Heilongjiang 150001, People’s Republic of China; c School of Materials, Sun Yat-sen University, Guangzhou, Guangdong 510275, People’s Republic of China; d Hawaii Institute of Geophysics and Planetology, University of Hawaii at Manoa, Honolulu, HI 96822, USA; e Center for Advanced Radiations Sources, University of Chicago, Chicago, IL 60439, USA

**Keywords:** pressure-induced phase transition, negative linear compressibility, bulk moduli, isostructural phase transition

## Abstract

A series of *in situ* synchrotron X-ray diffraction (XRD) measurements were carried out, combined with first-principles calculations, to study structural phase transitions of selenium at high pressures and room temperature. Several phase transitions were observed, among which an isostructural phase transition was found at around 120 GPa for the first time. Evolved from the rhombohedral (space group *R* 3 *m*) structure (Se-V), the new phase (Se-V′) exhibited an interesting increase of lattice parameter *a* at pressures from 120 to 148 GPa, known as negative linear compressibility (NLC). The discovery of NLC behavior observed in this work is mainly attributed to the accuracy and fine steps controlled by the membrane system for *in situ* XRD data collected with an exposure time of 0.5 s. After 140 GPa, a body-centered cubic (b.c.c.) structure Se-VI (space group *Im* 3 *m*) was formed, which remains stable up to 210 GPa, the highest pressure achieved in this study. The bulk moduli of phases Se-V, Se-V′ and Se-VI were estimated to be 83 ± 2, 321 ± 2 and 266 ± 7 GPa, respectively, according to the *P*–*V* curve fit by the third-order Birch–Murnaghan equation of state. The Se-V′ phase shows a bulk modulus almost 4 times larger than that of the Se-V phase, which is mainly due to the effect of its NLC. NLC in a higher pressure range is always more significant in terms of fundamental mechanism and new materials discovery, yet it has barely been reported at pressures above 100 GPa. This will hopefully inspire future studies on potential NLC behaviors in other materials at ultra-high pressure.

## Abstract 2

Structural evolutions of selenium on strong compression are still not fully understood, therefore *in situ* X-ray diffraction measurements up to 210 GPa were carried out. An anomalous negative linear expansion was observed at pressures from 120 to 148 GPa, accompanied by a new isostructural phase transition.

## 1. Introduction

Selenium, as well as other elements in the main group VIA, has been extensively studied in the field of materials science owing to its unique properties such as high photoconductivity, nonlinear optical response, large piezoelectricity and thermoelectric effect (Jafar *et al.*, 2016 ▸; Saleh *et al.*, 2017 ▸; Dieulesaint & Royer, 1982 ▸; Henkels & Maczuk, 1953 ▸). In addition, its intriguing inner structural behavior under high pressure which leads to a rich sequence of pressure-induced phases attracts numerous studies focusing on selenium under high pressure (Akahama *et al.*, 2021 ▸, 1993 ▸, 1992*a* ▸; Brazhkin *et al.*, 1992 ▸; Krüger & Holzapfel, 1992 ▸; Nishikawa *et al.*, 1996 ▸, 1993 ▸; Pal *et al.*, 2015 ▸; Parthasarathy & Holzapfel, 1988 ▸; Hejny & McMahon, 2004 ▸; Bandyopadhyay & Ming, 1996 ▸; Dai *et al.*, 2011 ▸). The rich pressure-induced phase transitions in selenium and other VIA family elements can be attributed to their unshared electron pairs which govern interplay of the intra- and inter-chain interactions and their sensitivity under pressure (Li *et al.*, 2013 ▸; Nagata & Miyamoto, 1993 ▸). Despite plenty of intensive studies of selenium and other VIA elements under pressure performed in the last half a century, the pressure-induced phase transition sequence (Parthasarathy & Holzapfel, 1988 ▸; McMahon *et al.*, 2004 ▸; Akahama *et al.*, 1993 ▸), compressibility (Baughman *et al.*, 1998 ▸; Cairns & Goodwin, 2015 ▸; McCann *et al.*, 1972 ▸; Ren *et al.*, 2009 ▸; Keller *et al.*, 1977 ▸) and the dynamics of pressure-induced crystallization from amorphous Se (Yang *et al.*, 2007 ▸; Ye & Lu, 1998 ▸; Tanaka, 1990 ▸; Liu *et al.*, 2008 ▸; Brazhkin & Tsiok, 2017 ▸; Harrison, 1968 ▸; Akahama *et al.*, 1997 ▸) are still controversial.

To date, various pressure-induced phase-transition sequences have been reported on trigonal Se. Parthasarathy & Holzapfel (1988 ▸) reported the Se of the trigonal structure directly transformed to the monoclinic phase at 14 GPa, then to the tetragonal phase at 28 GPa. Akahama *et al.* (1993 ▸) reported that compressed trigonal Se experienced an intermediate phase from 14 to 23 GPa, and then transferred to a base-centered monoclinic phase at 23 GPa, *i.e.* trigonal → intermediate phase → base-centered monoclinic sequences. In the higher-pressure range, the phase transition from an incommensurately modulated monoclinic structure to a β-Po type structure at 40–60 GPa was observed, whereas McMahon *et al.* (2004 ▸) suggested this transition occurs at 82 GPa by extrapolating the monoclinic structural parameters, and Degtyareva *et al.* (2005*a* ▸) experimentally observed this phase transition at 80 GPa. So far, about seven selenium allotropes related to high-pressure and high-temperature conditions have been reported experimentally (Degtyareva *et al.*, 2005*b* ▸, 2007 ▸; Akahama *et al.*, 2021 ▸). Therefore, the pressure-induced phase transition sequence of trigonal Se should be as follows: trigonal phase (Se-I, space group *P*3_1_21) → *C*-face-centered monoclinic phase (Se-II, space group *P*2_1_/*c*, 14 GPa) → metallic base-centered monoclinic phase (Se-III, space group *C*2/*m*, 23 GPa) → incommensurate monoclinic phase (Se-IV 28 GPa) → rhombohedral phase with β-Po structure (Se-V, ∼80 GPa) → b.c.c. phase (Se-VI, 140 GPa). Phase Se-VI was reported to be stable up to 317 GPa, the highest recorded experimental pressure value for Se to the best of our knowledge (Akahama *et al.*, 2021 ▸).

Theoretical studies based on density-functional calculations showed a structural phase transition for Se from the b.c.c. to the f.c.c. structure occurs around 260 GPa (Rudin *et al.*, 2001 ▸). Other calculations indicated that Te transfers from a b.c.c. structure to an intermediate structure at 100 GPa, then to an f.c.c. structure at 255 GPa (Sugimoto *et al.*, 2014 ▸). Given the previous calculations and the similarity between Te and Se in terms of pressure-induced phase transition sequences, it is important to explore the possible phase transitions of Se experimentally. In the current work, pressure-induced phase-transition sequences in selenium up to 210 GPa are explored.

Among the inconsistencies with pressure-induced phase-transition sequences, the specific phase transition from the Se-V phase to the Se-VI phase has drawn much theoretical and experimental attention. Though Akahama *et al.* (2021 ▸, 1993, 1992*b* ▸) reported the Se-V to Se-VI transitional pressure is around 140 GPa in experiment, many calculations indicate there might be a phase transition around 120, 110 GPa or even as low as 95 GPa. Rudin *et al.* (2001 ▸) predicted the phase transition occurrs at 120 GPa based on density-functional theory (DFT). This is consistent with calculations carried out by Otani & Suzuki (2001 ▸), which revealed the transition pressure at 110 GPa based on the first-principles calculations performed on electronic structures of Se-V and Se-VI phases. Similar results of phase transitional pressure at 110 GPa were reported by Nishikawa *et al.* (1993 ▸), who calculated the related band-structure and total energy. However, using the full-potential linearized-augmented-plane-wave method, Geshi *et al.* (1998 ▸) revealed the transition was at a lower pressure of 95 GPa. Obviously, the controversies are not only between calculation and experiment, but also between various calculations themselves. In the current experiment, a phase transition around 120 GPa is observed and its interesting behavior at pressure above 120 GPa will be discussed.

Note that Akahama *et al.* (1992*b* ▸) observed some discontinuity of the pressure dependence of bond length at about 120 GPa, which might propose a possible second-order phase transition. However, in their recent work, this issue was left unaddressed (Akahama *et al.*, 2021 ▸). In the current work, a clear discontinuity of volume change with increasing pressure was observed via *in situ* X-ray diffraction (XRD) around 120 GPa, accompanied with a linear expansion in lattice parameter *a*. Compared with earlier works, where negative linear expansion (NLE) in materials was only observed under comparatively lower pressures, this work presents NLE in Se at pressures over 100 GPa, which is of great interest and significance in terms of new materials discovery.

## 2. Experimental procedure and calculation details

The trigonal Se sample (99.99% purity) was loaded in the hole of the rhenium gasket and compressed between the membrane-driven diamond anvil cell (DAC). Powder XRD data were collected at GSECARS and HPCAT, Advanced Photon Source, Argonne National Laboratory. Focused monochromatic beams of 0.4066 Å wavelength were used and the diffraction patterns were collected with a PILATUS 1M detector. Two-dimensional XRD patterns were integrated to create one-dimensional patterns using the *Dioptas* program (Prescher & Prakapenka, 2015 ▸). Gold and silicone oil were used as pressure marker and pressure transmitting media, respectively. The beveled diamond anvils with 70–300 µm culet diameter were used. Rhenium gaskets were pre-indented to a thickness of 20 µm and then drilled to make a sample chamber hole 30 µm in diameter. The sample of trigonal Se was ground, then loaded into the gasket hole along with a few several-micrometre-sized gold foils, and the silicone oil was added before sealing the chamber. The size of the X-ray spot was about 10 µm in diameter. The diffracted X-ray signal was detected with an exposure time of 0.5 s, while constant compression was applied by the membrane-controlled DAC system, with a compression rate at about 0.6 GPa s^−1^ in the chamber. Diffracted images were collected throughout the experiment, until the pressure in the sample chamber reached 210 GPa. Like the majority of high-pressure DAC experiments over 100 GPa, the DACs were broken in this run after the pressure reached 210 GPa. Therefore, no diffraction data were available during the decompression process and the questions of possible hysteresis and phase metastability ranges on pressure release remain unanswered.

XRD from both the selenium and gold were measured simultaneously by selecting a spot on the sample where both were present. This approach minimized the possible systematic errors due to pressure gradients and ensured accurate *in situ* pressure measurements. Pressures were calculated using the equation of state (EoS) for Au under pressure (Anderson *et al.*, 1989 ▸).

The first-principles calculation was performed using the plane wave DFT program *VASP* [Vienna ab initio simulation package (Kresse & Furthmüller, 1996 ▸)], where the electron–ion interactions of Se atoms were represented by the projector augmented wave (PAW) pseudopotential (Blöchl *et al.*, 1994 ▸). The exchange-correlation functional was described by the generalized gradient approximation (GGA) in the Perdew–Burke–Ernzerhof (PBE) parameterization (Perdew *et al.*, 1996 ▸). The calculations were computed with a 10 × 10 × 12 Monkhorst–Pack scheme (Monkhorst & Pack, 1976 ▸). The energy cut off was set at 550 eV. The geometry convergence criterion was set at 0.001 eV Å^−1^ for the maximal component of force and 0.01 GPa for stress.

## 3. Results and discussion

**Figure 1:**
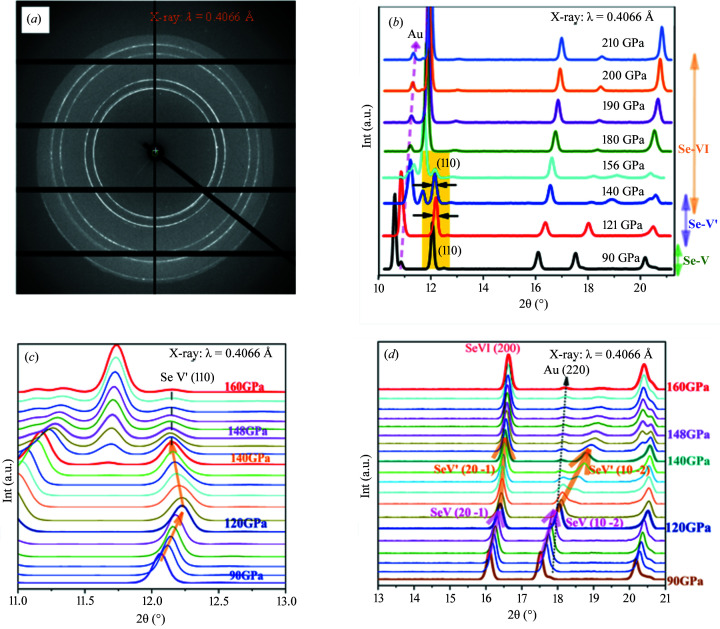
(*a*) 2D diffraction image of Se under 90 GPa pressure. (*b*) XRD patterns of Se compressed under selected pressures from 90 to 210 GPa at ambient temperature, evolving from Se-V to Se-V′, then to Se-VI. A zoomed-in view of (*b*) with more XRD patterns added between the plotted ones, from 90 to 160 GPa, of 2θ (*c*) 11 to 13°; (*d*) 13 to 21°.

The *in situ* XRD patterns of Se were collected under constant compression and short-time exposure, and these high-resolution synchrotron diffraction patterns were able to show minute changes in the structure. More than 4300 XRD 2D images were collected while the sample was compressed from ambient pressure to 210 GPa. Fig. 1 ▸(*a*) representatively shows one of the typical XRD images, under 90 GPa. The 2D images were integrated into 1D XRD patterns using *DIOPTAS* (Prescher & Prakapenka, 2015 ▸), shown in Fig. 1 ▸(*b*). Note that the patterns from 90 to 140 GPa match the hexagonal Se-V (β-Po-type rhombohedral) structure. With increasing pressure to 140 GPa, a new peak near 11.8° 2θ appears, indicating a phase transition to Se-VI (b.c.c. structure). This is consistent with previous works where the phase transition from Se-V to Se-VI was found at 140 GPa (Akahama *et al.*, 2021 ▸, 1993 ▸; Degtyareva *et al.*, 2005*a* ▸). On further compression, Se-VI remains stable with no other phase transitions observed from 140 to 210 GPa.

Interestingly, a closer look into the XRD patterns above 120 GPa reveals that the diffraction peak (110) of Se-V starts to shift towards a lower value of 2θ with increasing pressure, while other diffraction peaks are continually moving towards the larger value, as one would expect for elevated pressures. This trend is more clearly shown in Fig. 1 ▸(*c*). The shift is observed during compression from 120 to 148 GPa. According to the Bragg equation, this indicates the (110) peak moves towards a larger *d*-spacing value, which implies that the interplanar distance of (110) planes increases with pressure. Given the hexagonal interplanar spacing relationship with lattice parameters at a given plane 

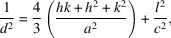

it can be concluded that lattice parameter *a* in hexagonal Se increases with pressure in this range. Fig. 1 ▸(*d*) shows interplanar spacing changes from other planes: peaks (201) and (102). From 90 to 120 GPa, both (201) and (102) peaks move towards larger 2θ values with higher pressure at relatively high rates. However, once the pressure is increased to above 120 GPa, (201) moves (towards larger 2θ values) at a decreased rate, while (102) moves at an increased rate. Considering the interplanar spacing relationship given above, one can deduce that once the pressure increases to above 120 GPa, the lattice along the *c* direction is more compressible whereas along *a* is the opposite case.

Noticeably, Akahama *et al.* (1993 ▸) also reported a relevant observation with Se at the same pressure region. They observed that when the Se-V phase was compressed to pressures above 120 GPa, the first-nearest neighbor bond length barely decreased with increasing pressure, while the second-nearest neighbor bond length decreased more drastically. However, there were not enough finely spaced data to obtain an accurate trend of the lattice parameter *a* change at this dedicated pressure range in their work. In the current work, with higher-resolution data as well as the fine control of exposure time and compression rate in this experiment, we were able to observe the abnormal increase of the distance between (110) planes, which implied that the first-nearest neighbor bond of a given Se atom increased with pressure during 120–148 GPa, rather than just ‘an unusual hardening’ as pointed by Akahama *et al.* (1993).

**Figure 2:**
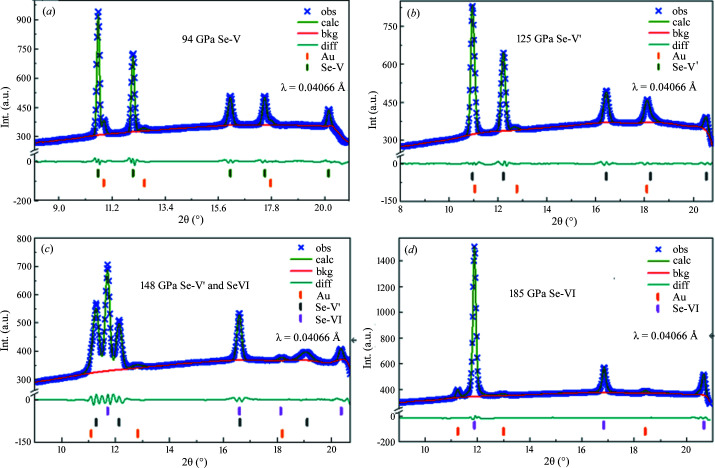
Representative Rietveld refined diffraction patterns and fitting results of (*a*) Se-V at 94 GPa, with *a* = 3.8687 (6) Å and *c* = 2.9086 (2) Å (*W*_R_ = 1.17%, χ^2^ = 25.6564, GOF = 0.23); (*b*) Se-V′ at 125 GPa with *a* = 3.8205 (18) Å and *c* = 2.7760 (3) Å (*W*_R_ = 0.94%, χ^2^ = 17.3023, GOF = 0.18); (*c*) Se-V′ with *a* = 3.8450 (30) Å and *c* = 2.6371 (6) Å, Se-VI with *a* = 2.8191 (6) Å at 148 GPa (*W*_R_ = 1.06%, χ^2^ = 20.719, GOF = 0.21); and (*d*) Se-VI at 185 GPa, with *a* = 2.7755 (5) Å (*W*_R_ = 2.12%, χ^2^ = 87, GOF = 0.42).

In order to take a closer look at the change in lattice parameters, Rietveld fitting was employed to extract lattice parameters using *GSAS-II* (Toby & Dreele, 2013 ▸). Some of the patterns with Rietveld refinement at different pressures are as shown in Fig. 2 ▸.

**Figure 3:**
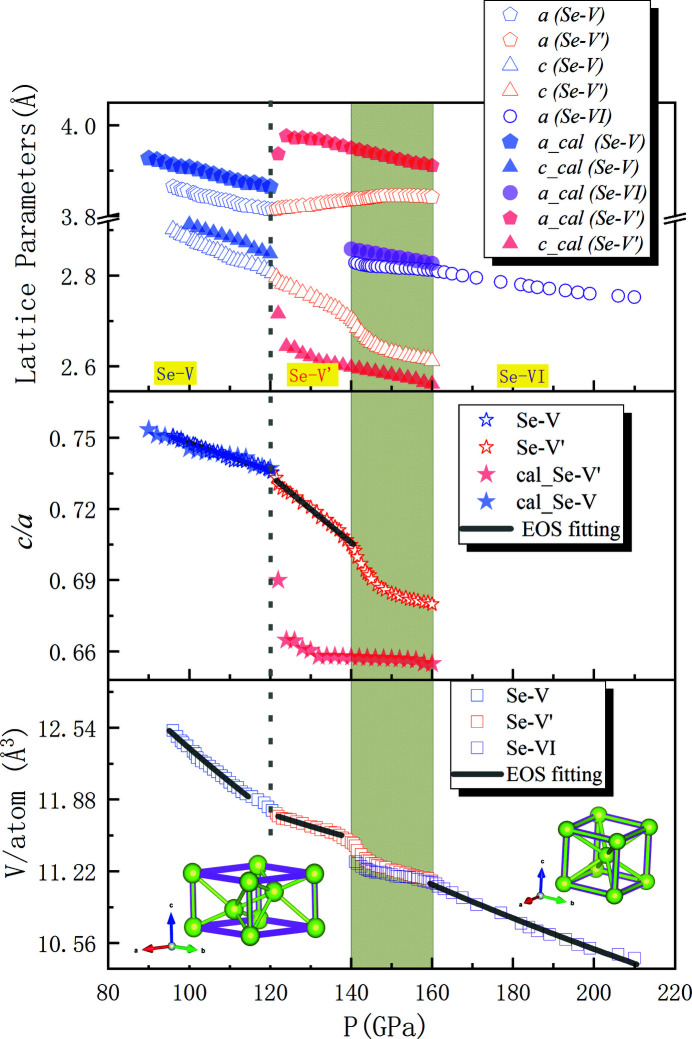
Pressure dependence of lattice parameters *a* and *c*, and atomic volume *V* from 90 to 210 GPa. The open symbols represent experimental data in this work, the solid symbols represent the calculated lattice constants *a* and *c*, and the *c*/*a* ratio, respectively. The b.c.c. structure remains stable up to 210 GPa, no new phase transition was observed from 140 to 210 GPa. The phases Se-V′ and Se-VI coexist from 140 to 160 GPa.

Lattice parameters *a* and *c*, the *c*/*a* ratio, and atomic volume versus pressure are plotted, as shown in Fig. 3 ▸. It is evident that below 120 GPa, *a*, *c* and *c*/*a* decrease with increasing pressure. However, an increase of the lattice parameter *a* occurs once the pressure exceeds 120 GPa, which corresponds with the shift of diffraction peak (110) to lower angles starting from 120 GPa. This increase lasts until the pressure reaches 148 GPa, during which *a* is calculated to increase by 2.22%. Such a linear increase in lattice parameters and the vast difference in bulk modulus indicate an intermediate phase existing between 120 and 148 GPa, referred to as Se-V′ here.

Accompanied with the increase of lattice parameter *a*, lattice parameter *c* decreases at a higher rate, which guarantees the continuous volume decrease. It is obvious from the pressure dependence of atomic volume that the increase of *a* slows down the volume decrease to some extent. It is worth noting that the increase of *a* corresponds with the slower rate of volume decrease. The discontinuity of pressure dependence of atomic volume at 120 GPa and a different slope in the EoS fitting curve from 120 to 148 GPa indicate a phase transition happening with a lower compressibility of Se under the pressure from 120 to 148 GPa. This suggests an isostructural phase transition around 120 GPa, followed by another phase transformation to a b.c.c. structure (Se-VI) at 140 GPa. In Fig. 3 ▸, the shaded area in the graph represents the region where the Se-V′ and Se-VI phases coexist.

**Figure 4:**
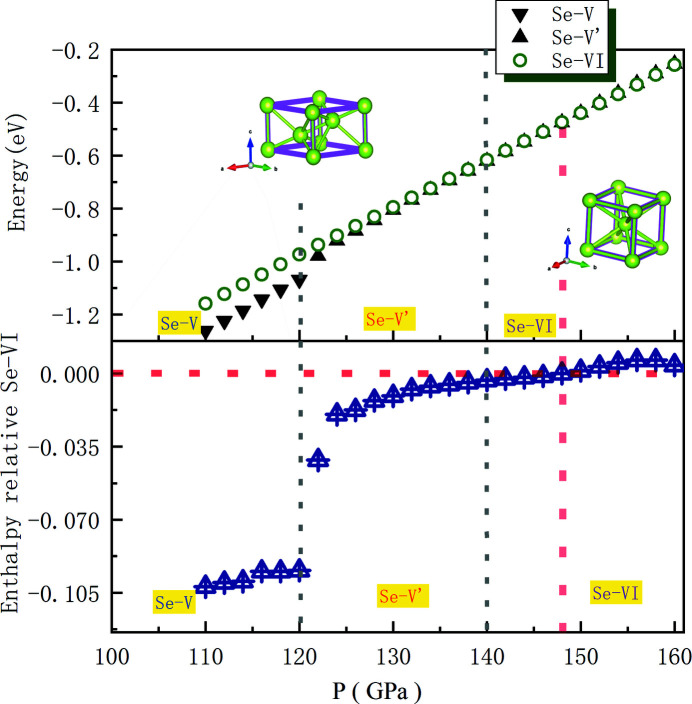
Energy of Se-V′ and Se-VI (relative to the Se-V structure) as a function of pressure from 110 to 160 GPa.

In order to have a comprehensive understanding of such phenomenon, DFT calculations were carried out to study Se under high pressure. From the DFT calculations, *a* shows an increase above 120 GPa, which is consistent with the experiment, and then, a decrease with compression as shown in Fig. 3 ▸. Regardless of the absolute value difference between calculations and experimental data, the trend of *c*/*a* ratio is in fair agreement with the experimental data, which supports the fact that Se-V has experienced an isostructural phase transition at around 120 GPa. We further plotted the energy of Se-V′ and Se-VI (relative to the Se-V structure) as a function of pressure in Fig. 4 ▸. The structure of Se-V′ remained stable up to 148 GPa, while Se-VI was calculated to be more stable at pressures higher than 148 GPa. However, in the experiment, Se-V′ transformed into Se-VI at about 140 GPa. Such a small discrepancy might be due to the limitation of the pseudopotential used in the *VASP* code, which is beyond the scope of our current work. Noticeably, the energy of the Se-VI phase relative to the Se-V structure is drastically reduced at 120 GPa, which indicates a huge adjustment of the lattice to accelerate the transition to the b.c.c. structure. Overall, the DFT calculation values are in adequate agreement with our experimental results, which have revealed the Se-V to Se-V′ isostructural phase transition takes place around 120 GPa whereas the Se-V′ to Se-VI structural phase transition takes place around 140 GPa.

The volume–pressure relationship was fitted according to the third-order Birch–Murnaghan EoS (Singh, 2005 ▸; Jeanloz, 1988 ▸; Birch, 1947 ▸), as shown on Fig. 3 ▸. The fitting results provide a value for the bulk modulus *B*_0_ as 83 ± 2 GPa for Se-V, 321 ± 2 GPa for Se-V′ and 266 ± 7 GPa for Se-VI. All the other fitting parameters are shown in Table 1 ▸. Recently, Akahama *et al.* (2021 ▸) reported the bulk modulus of Se-VI as 290 GPa using the diffraction data up to 317 GPa. Generally, more compression data covering a wider pressure region could yield a more precise bulk modulus. More *P*–*V* data, especially at a higher-pressure range between 210 and 317 GPa would achieve a better estimation of the bulk modulus. For easy comparison, the derivative of the bulk modulus *B*′ was fixed at 3.2 during the fitting in our case, the same as the value of the Se-VI phase obtained in the recent report by Akahama *et al.* (2021 ▸). However, due to the flattening in the *P*–*V* curve near 300 GPa, their reported bulk modulus of Se-VI is slightly higher than the value from the current report. The other difference is the bulk modulus of Se-V, which they reported as 205 GPa, whereas in the current report, a smaller value of 83 ± 2 GPa for Se-V, and a larger value of 266 ± 7 GPa for Se-V′ are reported. This could be due to the combined effect since *P*–*V* data from phase Se-V and Se-V′ were not fitted separately in their report.

Interestingly, Se-V′ has the biggest bulk modulus and the smallest compressibility among the three phases, which indicates Se-V′ is the least compressible. As mentioned above, in Fig. 3 ▸ the increase of the lattice parameter *a* is accompanied by a decrease of volume at a reduced rate, which leads to a least compressible intermediate phase from 120 to 148 GPa. The increase of lattice constant *a* contributes to relaxation in the distortion from hexagonal Se-V, β-Po-type rhombohedral structure, to Se-V′, which accelerates the structural evolution from Se-V to the b.c.c. structure Se-VI. Geshi *et al.* (1998 ▸) reported a similar phenomenon that the compressibility increased in the b.c.c. structure when compared with that in the previous phase, but no explanation was given. They also conducted calculations which suggested a phase transition around 95 GPa but wrongly assigned this phase transition as that observed experimentally at 140 GPa (Akahama *et al.*, 1993 ▸).

Similar to the increase of lattice parameter *a* observed in Se-V′ at high pressure, similar linear expansion has been observed in other materials under pressure, especially with structures like soft porous frameworks (Sobczak *et al.*, 2020 ▸; Cai *et al.*, 2015 ▸; Cai & Katrusiak, 2014 ▸), ferroelastics, tilting networks and helices (Cairns & Goodwin, 2015 ▸). This counterintuitive high-pressure behavior is usually referred to as negative linear compressibility (NLC). It arises whenever volume reduction can be coupled to linear expansion.

Notably, NLC has only been observed in materials under comparatively lower pressures. For example, NLC was found at pressures lower than 14 GPa in selenium with a helical structure, referred to as Se-I (Cairns & Goodwin, 2015 ▸; McCann *et al.*, 1972 ▸; Keller *et al.*, 1977 ▸; Akbarzadeh *et al.*, 1993 ▸). The rare occurrence of NLC at much higher pressures (*i.e.* >100 GPa) is uncommon. As shown in this work, the NLC is observed in hexagonal selenium with a β-Po-type rhombohedral structure between 120 and 148 GPa.

**Figure 5:**
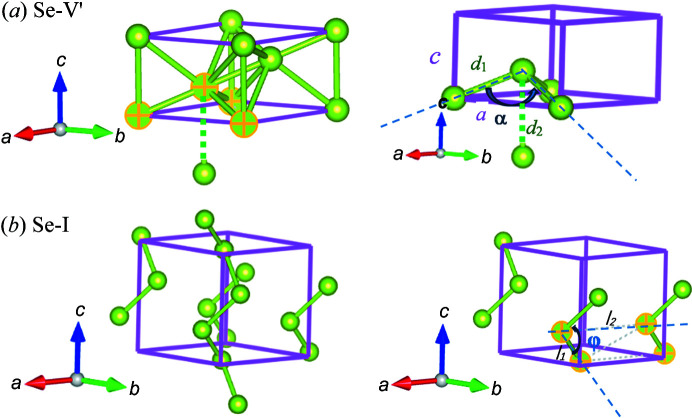
Schematic of geometrical interpretation of negative linear compressibility in the structures of (*a*) Se-V′ and (*b*) Se-I.

The marked four atoms in the β-Po structural unit cell from Fig. 5 ▸(*a*) form a tetrahedron with lattice parameter *a* as sides of the base face; *d*_1_ and *d*_2_ are bond lengths from first-nearest and second-nearest neighbors, respectively; and α is the angle formed by atoms 1, 2 and 3, as depicted in Fig. 5 ▸(*a*). From 120 to 148 GPa, with increasing pressure both *d*_1_ and *d*_2_ (corresponding to lattice parameter *c*) decrease, while α increases, which results in a shorter and flatter tetrahedron. Such a geometrical change of structure leads to an increase of lattice parameter *a* under pressure. Note, in this process, *d*_1_ decreases much slower compared with *d*_2_, and its decreasing rate reduces rapidly with compression. This indeed accelerated the transformation from a β-Po-type rhombohedral structure to a cubic structure.

In terms of the NLC phenomenon with Se-I, which has a trigonal structure consisting of helices, *c* was observed to increase with elevating pressure, while *a* decreased. In this structure, the bonds between helices are much weaker than those within a specific helix. As a result of this anisotropy, the bond length in the *a* direction decreases while it increases in the *c* direction. The marked atoms in Fig. 5 ▸(*b*) form a parallelogram with two sides labeled *l*_1_ and *l*_2_, between which the smaller angle is marked as φ. Therefore, an increase of *l*_1_ indicates an increase of *c* and vice versa, and *l*_2_ corresponds to *a*. With increasing pressure, *l*_2_ (parameter *a*) decreases rapidly, while both φ and *l*_1_ (parameter *c*) increase. As a result, the pressure-induced densification of Se-I demonstrates preferential compression of *a* at the expense of some expansion in *c*.

Table 2 ▸ shows previous works on NLC observations in Se, including Se-I, and the current work on the Se-V′ structure. In the high-pressure structure, the absolute value of linear compressibility β in Se-V′ is about an order of magnitude smaller than that of Se-I. Cairns & Goodwin (2015 ▸) summarized some other materials with tilting network structures that exhibited NLC behavior under pressure, usually due to some correlated polyhedral tilts. This is similar to the mechanism of NLC in Se-V′ discovered in this work.

Compared with the NLC occurring in Se-I, the NLC observation in Se-V′ is more remarkable because the latter happened at pressures over 100 GPa. Considering the analogy of behavior and structure dependence among the elements in the VIA group under pressure, there is great promise in exploring possible NLC occurrences in S and Te under ultra-high-pressure conditions. Interestingly, Hsueh *et al.* (2000 ▸) reported the NLC phenomenon in Te with *c* at pressures <5 GPa, which can be related to that of selenium in Se-I within 14 GPa. With this discovery of NLC in Se-V′ at >100 GPa, there are more potential NLC phenomena in elements S and Te to explore under strong compression. This NLC behavior might be a universal feature in many elements under ultra-high-pressure conditions.

Note that the pressure media used in this work, silicone oil, does not guarantee the hydro­static pressure environment. Besides, the potential effects from varying compression rate would fine tune sample strain and stress state (Konôpková *et al.*, 2015 ▸). A better pressure media like He or Ne is suggested for future works with various fast compression rates to substantially minimize the role of uniaxial stress and maximize the potential strain rate effect on this dedicated research topic.

## 4. Conclusions

In this work, selenium was compressed up to 210 GPa. An isostructural phase transition from Se-V to Se-V′ was observed for the first time around 120 GPa, and the newly discovered Se-V′ phase was estimated to have the largest bulk modulus of 321 ± 2 GPa, compared with that of Se-V (83 ± 2 GPa) and Se-VI (266 ± 7 GPa). The largest bulk modulus of Se-V′ indicates its lowest compressibility among the three phases, which can be attributed to the occurrence of NLC during compression. Considering that NLC at higher pressure is always more significant in terms of fundamental mechanism and new materials discovery, and that it has barely been reported at such high pressures above 120 GPa, this discovery inspires future studies into NLC behavior in new materials at higher pressures. NLC behavior observed in selenium in this work is mainly attributed to the accuracy and fine steps of *in situ* XRD data collected at 0.5 s intervals. Such high-resolution measurements make it possible to map the evolution of sample structure at a continuously increasing pressure. The discoveries of the new phase Se-V′ and its NLC behavior in selenium, a material considered one of the most studied elements in high-pressure research, illustrate a promising future where better understandings can be achieved by taking advantage of rapidly improving experimental techniques.

## Supplementary Material

Lattice parameters of Se in the pressure range 90-210 GPa. DOI: 10.1107/S2052252522000252/zx5024sup1.pdf

## Figures and Tables

**Table 1:** The EoS parameters of selected Se high-pressure phases Reference volume *V*_0_, bulk modulus *B*_0_ and the derivative of the bulk modulus with respect to pressure *B*_0_′, obtained from the least-squares fitting of the experimental data according to the third-order Birch–Murnaghan EoS. Values in brackets are estimated standard uncertainties and refer to the least significant digit; they reflect only the uncertainties derived from fitting *V* versus *P* points to the EoS.

Phases	*V*_0_ (Å^3^)	*B*_0_ (GPa)	*B*_0_′ (GPa)
Se-V	20.1 (3)	83 (2)	3.77 (6)
Se-V′	17.7 (1)	321 (2)	3.2 (Fixed)
Se-VI	15.76 (2)	266 (7)	3.2 (Fixed)

**Table 2:** Linear compressibilities of selenium Compressibilities of Se-I summarized from previous reported works and Se-V′ from this work.

Se phase	β_*a*_ (TPa^−1^)	β_*c*_ (TPa^−1^)	Structure	*P* (GPa)
Se-I (McCann *et al.*, 1972 ▸; Cairns & Goodwin, 2015 ▸)	12.00 (6)	−2.50 (4)	Trigonal	0–14
Se-I (Keller *et al.*, 1977 ▸)	15.97 (2)	−3.18 (1)	Trigonal	0–9.98
Se-V′†	−0.29 (2)	1.55 (1)	β-Po-type rhombohedral	121.5–148

† Calculated using *PASCal* (Cliffe & Goodwin, 2012 ▸) based on experimental lattice parameters.
