# Supplementary material for: Negative linear compressibility in Se at ultra-high pressure above 120 GPa
Source: IUCrJ. 2022 Feb 1;9(Pt 2):253–60. doi: 10.1107/S2052252522000252 (PMC8895011; doi:10.1107/S2052252522000252)
Supplement: Supplementary file 1 [file m-09-00253-sup1.pdf]

IUCrJ

Volume 9 (2022)

Supporting information for article:

Negative linear compressibility in Se at ultra-high pressure above 120 GPa

Shuhua Yuan, Luhong Wang, Sheng-cai Zhu, Fuyang Liu, Dongzhou Zhang, Vitali B. Prakapenka, Sergey Tkachev and Haozhe Liu

Table S1

Lattice parameters of the Se at pressure range of 90-210 GPa.

P	Se-V						Se-V'						c/a		Se-VI			
pressure (GPa)	a (Se-V)	sigma	c (Se-V)	sigma	Volume (Å3)	sigma	a (Å)	sigma	c (Å)	sigma	Volume (Å3)	sigma	Se-V	Se-V'	a (Å)	sigma	Volume (Å3)	sigma
94	3.8687	0.0006	2.9086	0.0002	12.5668	0.0016							0.7518					
96	3.8650	0.0005	2.9032	0.0010	12.5196	0.0025							0.7511					
97	3.8610	0.0012	2.8950	0.0003	12.4583	0.0030	--		--		--		0.7498	--				
98	3.8580	0.0003	2.8900	0.0001	12.4174	0.0008					--		0.7491	--				
98.5	3.8570	0.0018	2.8880	0.0003	12.4024	0.0043	--		--		--		0.7488	--	--			
99.5	3.8550	0.0010	2.8840	0.0008	12.3724	0.0033	--		--		--		0.7481	--	--			
101	3.8510	0.0022	2.8780	0.0011	12.3210	0.0063	--		--		--		0.7473	--	--			
101.5	3.8495	0.0008	2.8760	0.0006	12.3029	0.0026	--		--		--		0.7471	--	--			
102	3.8470	0.0031	2.8710	0.0007	12.2655	0.0076	--		--		--		0.7463	--	--			
103	3.8440	0.0029	2.8680	0.0008	12.2336	0.0073	--		--		--		0.7461	--	--			
104	3.8430	0.0006	2.8650	0.0001	12.2145	0.0014	--		--		--		0.7455	--	--			
105	3.8420	0.0011	2.8600	0.0009	12.1868	0.0036	--		--		--		0.7444	--	--			
106	3.8400	0.0017	2.8560	0.0025	12.1571	0.0071	--		--		--		0.7438	--	--			
107	3.8370	0.0007	2.8550	0.0021	12.1339	0.0045	--		--		--		0.7441	--	--			
108	3.8350	0.0029	2.8500	0.0013	12.1000	0.0079	--		--		--		0.7432	--	--			
109	3.8345	0.0003	2.8450	0.0012	12.0756	0.0023	--		--		--		0.7420	--	--			
110	3.8320	0.0009	2.8400	0.0007	12.0387	0.0029	--		--		--		0.7411	--	--			
111	3.8300	0.0013	2.8350	0.0020	12.0049	0.0055	--		--		--		0.7402	--	--			
112	3.8260	0.0021	2.8330	0.0004	11.9714	0.0049	--		--		--		0.7405	--	--			

P	Se-V						Se-V'						c/a		Se-VI			
pressure (GPa)	a (Se-V)	sigma	c (Se-V)	sigma	Volume (Å3)	sigma	a (Å)	sigma	c (Å)	sigma	Volume (Å3)	sigma	Se-V	Se-V'	a (Å)	sigma	Volume (Å3)	sigma
113	3.8240	0.0015	2.8310	0.0006	11.9505	0.0040	--		--		--		0.7403	--	--			
114	3.8222	0.0011	2.8286	0.0013	11.9291	0.0041	--		--		--		0.7400	--	--			
115.5	3.8210	0.0006	2.8250	0.0001	11.9064	0.0014	--		--		--		0.7393	--	--			
117	3.8190	0.0001	2.8210	0.0015	11.8771	0.0023	--		--		--		0.7387	--	--			
118.5	3.8170	0.0014	2.8150	0.0008	11.8395	0.0040	--		--		--		0.7375	--	--			
119.5	3.8150	0.0017	2.8110	0.0002	11.8103	0.0038	--		--		--		0.7368	--	--			
120.5							3.8140	0.0001	2.8050	0.0016	11.7789	0.0024		0.7354	--			
122							3.8170	0.0008	2.7880	0.0012	11.7259	0.0033	--	0.7304	--			
123							3.8180	0.0002	2.7820	0.0030	11.7068	0.0046	--	0.7287	--			
124							3.8190	0.0003	2.7790	0.0005	11.7003	0.0013	--	0.7277	--			
125							3.8205	0.0018	2.7760	0.0003	11.6969	0.0041	--	0.7266	--			
126.5							3.8210	0.0005	2.7700	0.0011	11.6746	0.0026	--	0.7249	--			
128							3.8230	0.0019	2.7620	0.0002	11.6531	0.0041	--	0.7225	--			
130							3.8240	0.0001	2.7560	0.0004	11.6339	0.0008	--	0.7207	--			
131.5							3.8260	0.0010	2.7500	0.0009	11.6207	0.0033	--	0.7188	--			
132.5							3.8280	0.0004	2.7420	0.0002	11.5990	0.0011	--	0.7163	--			
134							3.8300	0.0007	2.7400	0.0004	11.6027	0.0020	--	0.7154	--			
135							3.8310	0.0001	2.7330	0.0010	11.5791	0.0016	--	0.7134	--			
136							3.8320	0.0002	2.7300	0.0008	11.5724	0.0015	--	0.7124	--			
137							3.8320	0.0008	2.7250	0.0012	11.5512	0.0033	--	0.7111	--			
138							3.8340	0.0006	2.7160	0.0004	11.5251	0.0018	--	0.7084	--			
139							3.8350	0.0001	2.7110	0.0006	11.5099	0.0010	--	0.7069	--			
140							3.8360	0.0005	2.7040	0.0016	11.4861	0.0033	--	0.7049	--			
140.5							3.8370	0.0002	2.7000	0.0006	11.4751	0.0012	--	0.7037	--			

P	Se-V						Se-V'						c/a		Se-VI			
pressure (GPa)	a (Se-V)	sigma	c (Se-V)	sigma	Volume (Å3)	sigma	a (Å)	sigma	c (Å)	sigma	Volume (Å3)	sigma	Se-V	Se-V'	a (Å)	sigma	Volume (Å3)	sigma
141							3.8370	0.0007	2.6950	0.0004	11.4539	0.0020		0.7024	2.8280	0.0005	11.3086	0.0060
141.5							3.8370	0.0006	2.6850	0.0012	11.4114	0.0028	--	0.6998	2.8265	0.0001	11.2906	0.0012
142.5							3.8380	0.0001	2.6750	0.0002	11.3748	0.0005		0.6970	2.8243	0.0007	11.2643	0.0084
143.5							3.8390	0.0014	2.6650	0.0003	11.3382	0.0032		0.6942	2.8225	0.0009	11.2427	0.0108
144							3.8390	0.0007	2.6600	0.0006	11.3169	0.0022		0.6929	2.8219	0.0010	11.2356	0.0119
144.5							3.8400	0.0004	2.6580	0.0004	11.3143	0.0013	--	0.6922	2.8210	0.0010	11.2248	0.0116
145							3.8410	0.0001	2.6520	0.0004	11.2946	0.0008	--	0.6904	2.8207	0.0008	11.2212	0.0094
146							3.8420	0.0003	2.6490	0.0008	11.2877	0.0017	--	0.6895	2.8207	0.0006	11.2212	0.0073
146.5							3.8430	0.0008	2.6440	0.0002	11.2723	0.0018	--	0.6880	2.8200	0.0004	11.2129	0.0051
147.5							3.8440	0.0003	2.6400	0.0046	11.2611	0.0071	--	0.6868	2.8200	0.0003	11.2129	0.0030
148							3.8450	0.0030	2.6371	0.0006	11.2546	0.0067	--	0.6859	2.8190	0.0006	11.2010	0.0072
150							3.8450	0.0004	2.6320	0.0050	11.2328	0.0079	--	0.6845	2.8180	0.0004	11.1890	0.0047
151							3.8450	0.0009	2.6300	0.0053	11.2243	0.0093	--	0.6840	2.8168	0.0005	11.1748	0.0060
152							3.8450	0.0002	2.6270	0.0002	11.2115	0.0007	--	0.6832	2.8165	0.0012	11.1712	0.0143
153.5							3.8450	0.0014	2.6250	0.0001	11.2029	0.0028	--	0.6827	2.8163	0.0008	11.1688	0.0095
154.5							3.8450	0.0002	2.6220	0.0060	11.1901	0.0089	--	0.6819	2.8160	0.0001	11.1652	0.0012
155.5							3.8440	0.0002	2.6210	0.0004	11.1800	0.0010	--	0.6818	2.8155	0.0004	11.1593	0.0048
156.5							3.8440	0.0007	2.6190	0.0008	11.1715	0.0025	--	0.6813	2.8142	0.0009	11.1438	0.0107
158							3.8440	0.0014	2.6170	0.0002	11.1630	0.0030	--	0.6808	2.8130	0.0002	11.1296	0.0024
159							3.8430	0.0003	2.6160	0.0010	11.1529	0.0019	--	0.6807	2.8125	0.0008	11.1237	0.0095
160							3.8420	0.0001	2.6120	0.0007	11.1301	0.0012	--	0.6799	2.8120	0.0007	11.1177	0.0083
161															2.8110	0.0005	11.1059	0.0059
161.5															2.8090	0.0014	11.0822	0.0166
163															2.8070	0.0003	11.0585	0.0035

P	Se-V						Se-V'						c/a		Se-VI			
pressure (GPa)	a (Se-V)	sigma	c (Se-V)	sigma	Volume (Å3)	sigma	a (Å)	sigma	c (Å)	sigma	Volume (Å3)	sigma	Se-V	Se-V'	a (Å)	sigma	Volume (Å3)	sigma
165															2.8040	0.0011	11.0231	0.0130
167.5															2.8000	0.0003	10.9760	0.0035
171															2.7945	0.0001	10.9114	0.0014
177															2.7860	0.0002	10.8122	0.0023
182															2.7800	0.0005	10.7425	0.0058
184															2.7770	0.0014	10.7077	0.0162
185															2.7756	0.0005	10.6910	0.0058
186															2.7740	0.0001	10.6731	0.0012
189															2.7710	0.0008	10.6385	0.0092
193															2.7670	0.0005	10.5925	0.0057
196															2.7630	0.0002	10.5466	0.0023
199															2.7600	0.0007	10.5123	0.0080
206															2.7550	0.0006	10.4553	0.0068
210															2.7520	0.0007	10.4211	0.0080
